# Supplementary material for: Photo-produced aromatic compounds stimulate microbial degradation of dissolved organic carbon in thermokarst lakes
Source: Nat Commun. 2023 Jun 21;14:3681. doi: 10.1038/s41467-023-39432-2 (PMC10284890; doi:10.1038/s41467-023-39432-2)
Supplement: Supplementary file 1 — Supplementary Information [file 41467_2023_39432_MOESM1_ESM.pdf]

Supplementary Materials for

Photo-produced aromatic compounds stimulate microbial degradation of dissolved organic carbon in thermokarst lakes

Jie Hu[1,2], Luyao Kang[1,2], Ziliang Li[1,2], Xuehui Feng[1,2], Caifan Liang[1,2], Zan Wu[1], Wei Zhou[1,2], Xuning Liu[1,2], Yuanhe Yang[1,2], Leiyi Chen[1*]

[1]State Key Laboratory of Vegetation and Environmental Change, Institute of Botany, Chinese Academy of Sciences, Beijing 100093, China
[2]University of Chinese Academy of Sciences, Beijing 100049, China

[*]**Corresponding author:** Dr. Leiyi Chen, tel.: + 86 10-6283 6632, E-mail: chenly@ibcas.ac.cn

This PDF file includes:

Supplementary Methods
Supplementary Figure 1 to Supplementary Figure 13
Supplementary Table 1 to Supplementary Table 5
Supplementary Reference

Supplementary Methods

Calculation of the rates of light absorption by chromophoric dissolved organic matter (CDOM) and its uncertainty

The rate of light absorption by CDOM ($Q_{a,\lambda}$) is a product of photon dose and the concentration of CDOM available to absorb the light. We calculated $Q_{a,\lambda}$ in the 10 thermokarst lakes (Supplementary Figure 10) in 2020 as follows[1]:

$$Q_{a,\lambda} = \int_{\lambda_{\min}}^{\lambda_{\max}} E_\lambda \left(1 - e^{-a_{\mathrm{CDOM},\lambda} \times z}\right) \frac{a_{\mathrm{CDOM},\lambda}}{a_{\mathrm{tot},\lambda}} d\lambda \quad (1)$$

where $\lambda_{\min}$ and $\lambda_{\max}$ are the minimum and maximum wavelengths of ultraviolet (UV) light absorbed by CDOM (280 nm and 400 nm, respectively). E_λ is the photon flux spectrum (mol photon m^{-2} nm^{-1}), which was predicted by the NREL SMARTS model (Simple Model of the Atmospheric Radiative Transfer of Sunshine, V.2.9.5)[2]. Specifically, since there were no meteorological stations near these 10 thermokarst lakes, we used the reanalysis data from satellites as the input data (*e.g.*, atmospheric pressure, relative humidity, ozone abundance, and aerosol optical depth) for the model (Supplementary Table 5). Nevertheless, due to the strong elevation effects on aerosols and water vapor[3,4], these reanalysis data would lead to certain uncertainties in estimating E_λ. The fraction of light absorbed by CDOM relative to other light-absorbing constituents, $a_{\mathrm{CDOM},\lambda}/a_{\mathrm{tot},\lambda}$, was often equal to 1 at all wavelengths[2]. We assumed that the path length of light (z) was equivalent to the average depth of the lake (ranging from 0.24 m to 0.68 m) in the field. In addition, to compare the E_λ between the thermokarst lakes on the Tibetan Plateau and the arctic lakes, the direct and diffuse photon fluxes of the Tibetan Plateau thermokarst lakes at noon (1300 hr) on the summer solstice (June

21) in 2020 (wavelength: 300 to 600 nm) (Supplementary Figure 9) were also predicted by the model.

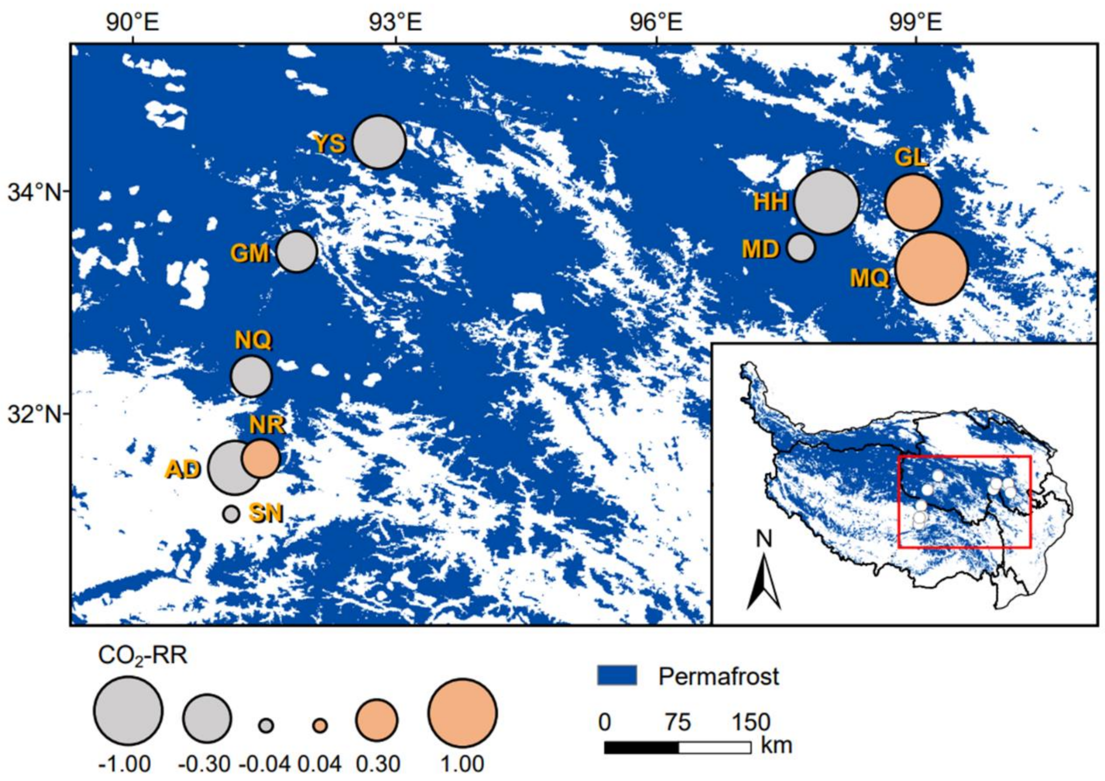

Supplementary Figure 1. Distribution map of lake sampling sites on the Tibetan Plateau. The bubble size indicates the magnitude of microbial respiration response to ultraviolet light exposure (CO_2-RR). The map of permafrost distribution is derived from [5]. The grey bubbles represent the negative sunlight effect, and the orange bubbles represent the positive sunlight effect. The ten sampling sites are Yushu (YS), Golmud (GM), Nagqu (NQ), Amdo County (AD), Nyainrong County (NR), Seni District (SN), Heihe River (HH), Golog Tibetan Autonomous Prefecture (GL), Madoi County (MD), and Maqên County (MQ). The map was created in ArcGIS Desktop 10.7 (ESRI, Redlands, CA, USA).

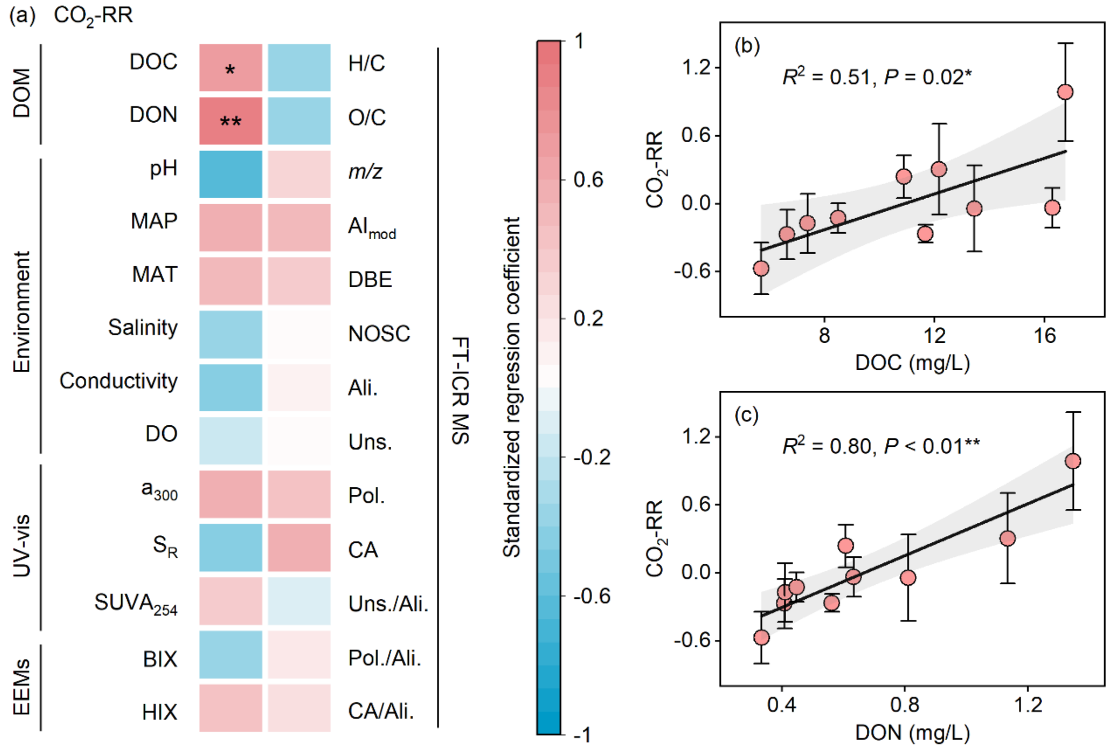

Supplementary Figure 2. Relationships of microbial respiration response to ultraviolet light exposure (CO_2-RR) with different water properties. (a) Standardized regression coefficients of these relationships, with the color indicating the strength and sign of the relationship. (b-c) Relationships between CO_2-RR and the significant predictors. The solid line and grey area represent the linear regression line and the 95% confidence interval, respectively. Dots with bars indicate means ± standard error (SE) (n = 3). The two-sided statistical tests indicate significant effects by *$P < 0.05$; **$P < 0.01$. DOC, dissolved organic carbon; DON, dissolved organic nitrogen; MAP, mean annual precipitation; MAT, mean annual temperature; DO, dissolved oxygen; a_{300}, the Naperian absorption coefficient at 300 nm; S_R, slope coefficient ratio; $SUVA_{254}$, the absorbance at 254 nm divided by DOC concentration; BIX, biological index; HIX, humification index; H/C, the ratio of the number of hydrogen atoms to the number of carbon atoms; O/C, the ratio of the number of oxygen atoms to the number

of carbon atoms; *m/z*, mass-to-charge ratio; AI_{mod}, modified aromaticity index; DBE, the double bond equivalence; NOSC, the nominal oxidation state of carbon; Ali., the number of aliphatic compounds; Uns., the number of highly unsaturated and phenolic compounds; Pol., the number of vascular plant-derived polyphenols; CA, the number of combustion-derived polycyclic aromatics; Uns./Ali., Pol./Ali. and CA/Ali. represent the ratio of Uns., Pol., and CA to Ali., respectively.

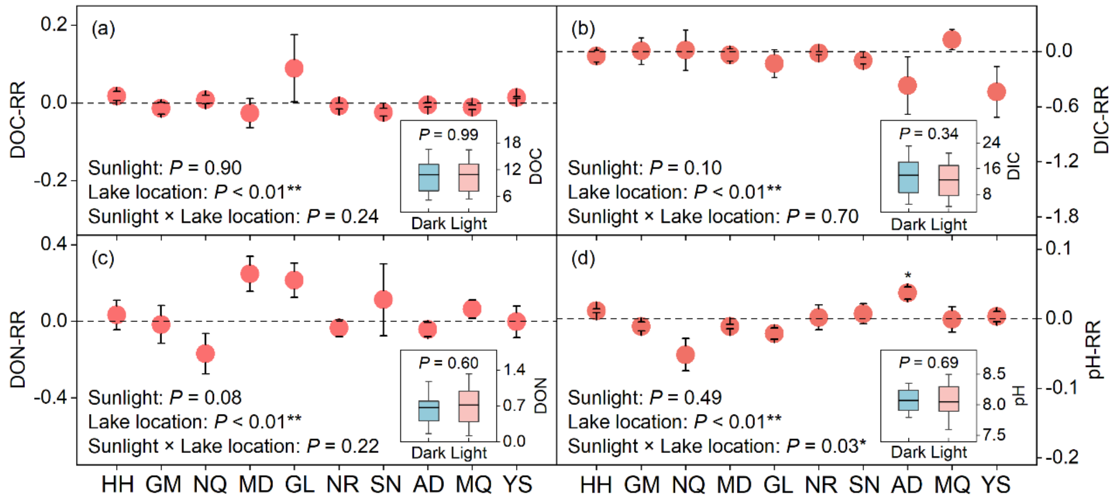

Supplementary Figure 3. Response ratios (RR) of water properties to sunlight exposure in the ten thermokarst lakes. The water properties include dissolved organic carbon (DOC, a), dissolved inorganic carbon (DIC, b), dissolved organic nitrogen (DON, c), and pH (d). The results of the two-way analysis of variance (ANOVA) of sunlight and lake location on water properties are shown in the lower-left corner. Dots with bars indicate means ± standard error (SE) (n = 3). The inserted box plot depicts the main effect of sunlight exposure on water properties using paired t-test. The ends of the boxes represent the 25th and 75th percentiles. The horizontal lines inside each box and the whiskers show the mean and 1.5 times the standard deviation. The black dashed line denotes the response ratio of zero. The two-sided statistical tests indicate significant effects by $*P < 0.05$; $**P < 0.01$. The ten sampling sites are Heihe River (HH), Golmud (GM), Nagqu (NQ), Madoi County (MD), Golog Tibetan Autonomous Prefecture (GL), Nyainrong County (NR), Seni District (SN), Amdo County (AD), Maqên County (MQ) and Yushu (YS).

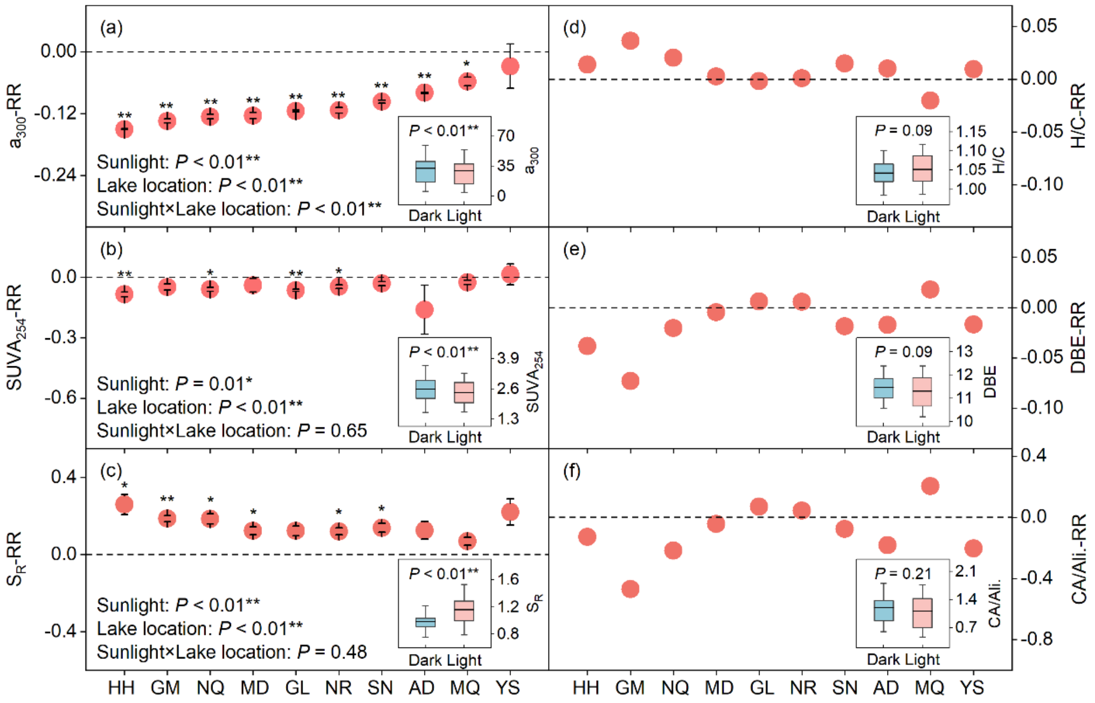

Supplementary Figure 4. Response ratios (RR) of dissolved organic carbon (DOC) chemistry to sunlight exposure in ten thermokarst lakes. DOC chemistry variables include the Naperian absorption coefficient at 300 nm (a_{300}, a), the absorbance at 254 nm divided by DOC concentration ($SUVA_{254}$, b), slope coefficient ratio (S_R, c), the ratio of the number of hydrogen atoms to the number of carbon atoms (H/C, d), the double bond equivalence (DBE, e), the ratio of the number of combustion-derived polycyclic aromatics to the number of aliphatic compounds (CA/Ali., f). The results of the two-way ANOVA of sunlight and lake location on water properties are shown in the lower-left corner. (a-c) Dots with bars indicate means ± standard error (SE) (n = 3). The inserted box plot depicts the main effect of sunlight exposure on the DOM chemistry using paired t-test. The ends of the boxes represent the 25th and 75th percentiles. The horizontal lines inside each box and the whiskers show the mean and 1.5 times the standard deviation. The black dashed line denotes the response ratio of zero. The two-sided statistical tests indicate significant effects by $*P < 0.05$; $**P < 0.01$. The ten

sampling sites are Heihe River (HH), Golmud (GM), Nagqu (NQ), Madoi County (MD), Golog Tibetan Autonomous Prefecture (GL), Nyainrong County (NR), Seni District (SN), Amdo County (AD), Maqên County (MQ) and Yushu (YS).

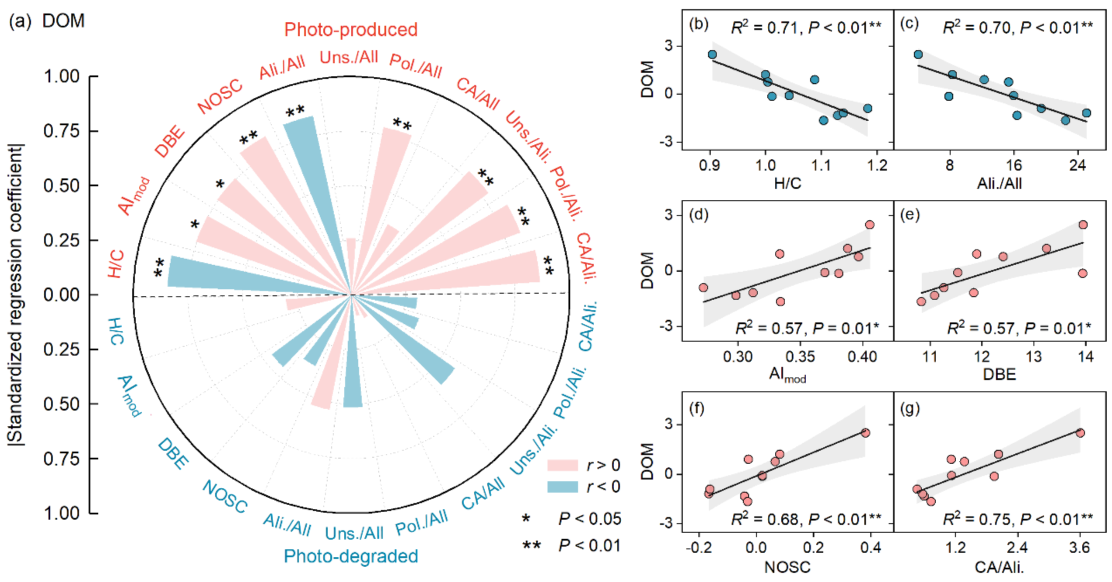

Supplementary Figure 5. Relationships of dissolved organic matter (DOM) content with the properties of photo-produced and photo-degraded compounds. (a) The length of the bar represents the absolute value of the standardized regression coefficient. The pink and blue bars indicate positive and negative relationships respectively. (b-g) Correlations of DOM content (represented as the first component of principal components analysis for dissolved organic carbon (DOC) and dissolved organic nitrogen content) with the properties of photo-produced compounds. The solid line and grey area represent the linear regression line and the 95% confidence interval, respectively. The two-sided statistical tests indicate significant effects by $*P < 0.05$; $**P < 0.01$. H/C, the ratio of the number of hydrogen atoms to the number of carbon atoms; AI_{mod}, modified aromaticity index; DBE, the double bond equivalence; NOSC, the nominal oxidation state of carbon; Ali./All, Uns./All, Pol./All, and CA/All represent the ratio of the number of molecular formulas of aliphatic compounds (Ali.), highly unsaturated and phenolic compounds (Uns.), vascular plant-derived polyphenols (Pol.) and combustion-derived polycyclic aromatics (CA) to the total number of molecular

formulas, respectively; Uns./Ali., Pol./Ali. and CA/Ali. represent the ratio of Uns., Pol., and CA to Ali., respectively.

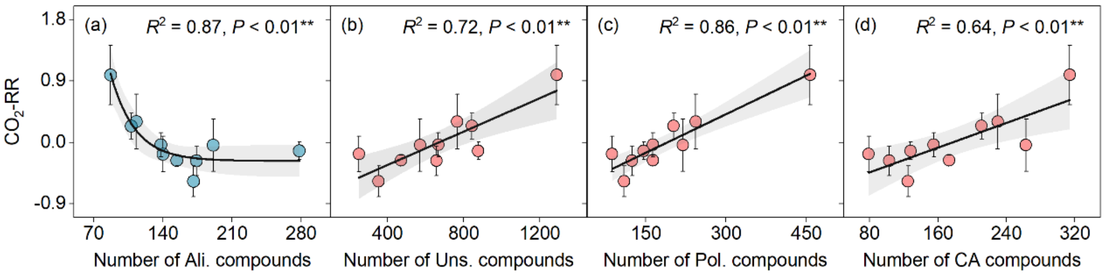

Supplementary Figure 6. Relationships between microbial respiration response to ultraviolet light exposure (CO_2-RR) and the number of photo-produced compounds. Correlations of CO_2-RR with the number of photo-produced aliphatic compounds (Ali., a), highly unsaturated and phenolic compounds (Uns., b), vascular plant-derived polyphenols (Pol., c) and combustion-derived polycyclic aromatics (CA, d). The solid line and grey area represent the linear regression line and the 95% confidence interval, respectively. Dots with bars indicate means ± standard error (SE) (n = 3). The two-sided statistical tests indicate significant effects by $*P < 0.05$; $**P < 0.01$.

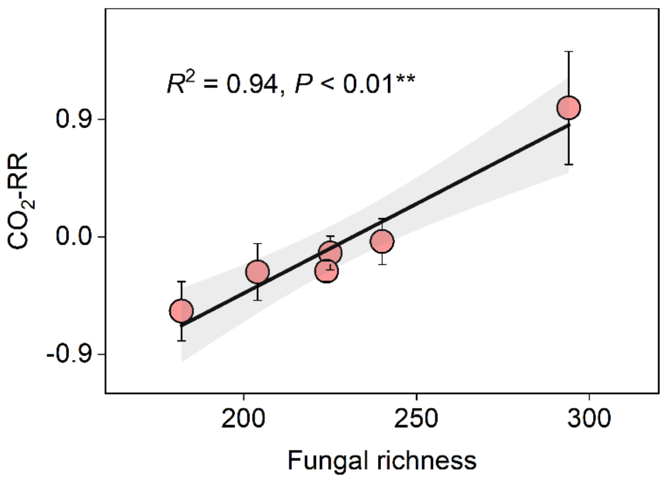

Supplementary Figure 7. Relationships of microbial respiration response to ultraviolet light exposure (CO_2-RR) with fungal richness. The solid line and grey area represent the linear regression line and the 95% confidence interval, respectively. Dots with bars indicate means ± standard error (SE) (n = 3). Because of insufficient sequence coverage, fungal data from 4 sampling sites (Golmud, Nyainrong County, Golog Tibetan Autonomous Prefecture, and Madoi County) were discarded. The two-sided statistical tests indicate significant effects by $^{**}P < 0.01$.

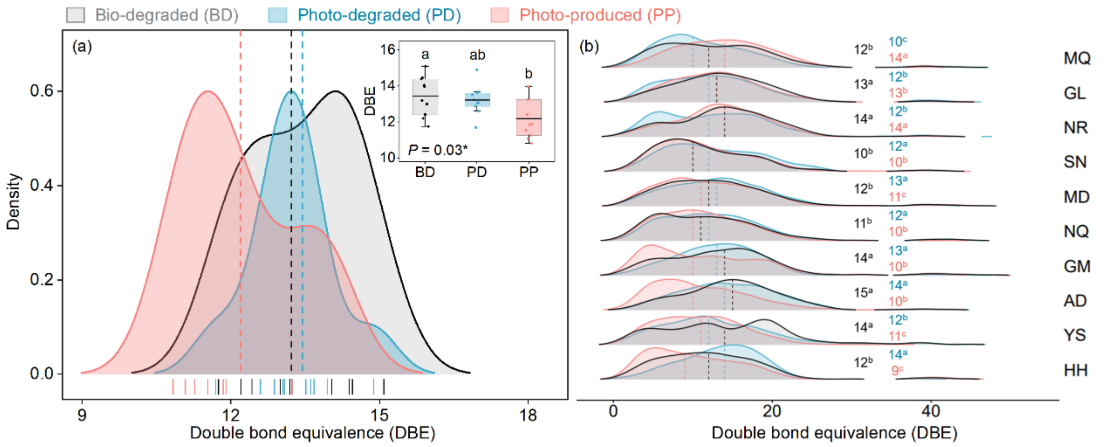

Supplementary Figure 8. Chemical properties of photo-produced (PP), photo-degraded (PD), and bio-degraded (BD) compound. The average double bond equivalence (DBE) of PP, PD, and BD of 10 lakes (**a**) and the AI_{mod} of each lake (**b**). The sampling sites of the ten lakes are Maqên County (MQ), Golog Tibetan Autonomous Prefecture (GL), Nyainrong County (NR), Seni District (SN), Madoi County (MD), Nagqu (NQ), Golmud (GM), Amdo County (AD), Yushu (YS) and Heihe River (HH). Insert box plots in panel (a) display the differences of DBE of PP, PD, and BD. The whiskers illustrate the 5th and 95th percentiles, and the ends of the boxes represent the 25th and 75th quartiles (interquartile range). The horizontal lines inside each box show the mean (n = 10). The data were analyzed using two-tailed one-way ANOVA, and significant effects are indicated as *$P < 0.05$, **$P < 0.01$. Based on two-sided tests for multiple comparisons by FDR corrections, different lowercase letters indicate significant differences among respective groups ($P < 0.05$). The black, blue, and pink number in panel (b) represents the median of the DBE of BD, PD, and PP compounds, respectively. A two-tailed Wilcoxon rank-sum test was used to assess the statistical significance in panel (b) with different letters indicating significant differences among the respective groups ($P < 0.05$).

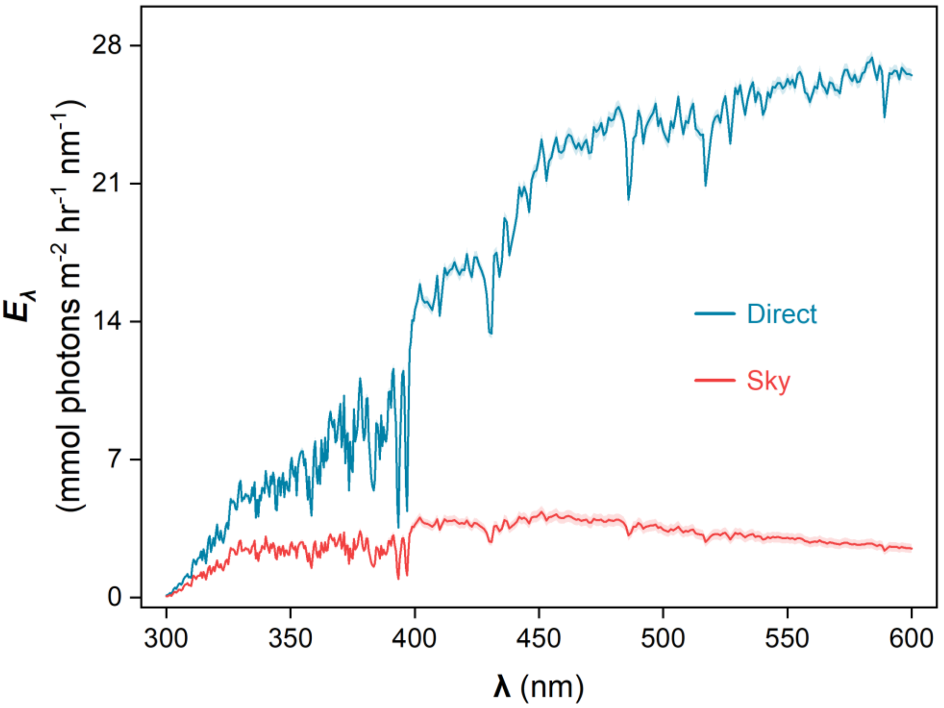

Supplementary Figure 9. Direct and diffuse (sky) photon flux spectrum (E_λ) above the 10 Tibetan Plateau thermokarst lakes water surface at 1300 hr local time on 21 June 2020. Solid lines represent mean curves, shaded areas represent means ± standard deviation (n = 10). The pink and blue lines indicate direct and diffuse photon flux densities, respectively.

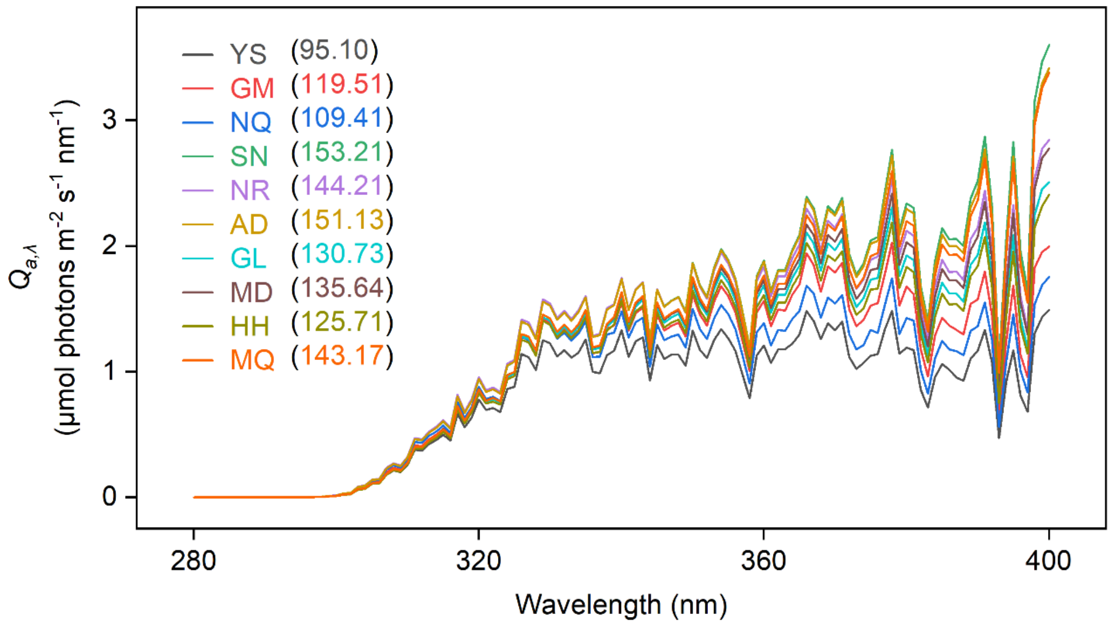

Supplementary Figure 10. Sunlight absorbed by chromophoric dissolved organic matter (CDOM) (*$Q_{a,\lambda}$*) in 10 thermokarst lakes. Different colors represent different lakes. The data in parentheses represent the integral value of sunlight absorbed by CDOM from 280 to 400 nm for each lake (μmol photons m^{-2} s^{-1}). The ten sampling sites are Yushu (YS), Golmud (GM), Nagqu (NQ), Seni District (SN), Nyainrong County (NR), Amdo County (AD), Golog Tibetan Autonomous Prefecture (GL), Madoi County (MD), Heihe River (HH), and Maqên County (MQ).

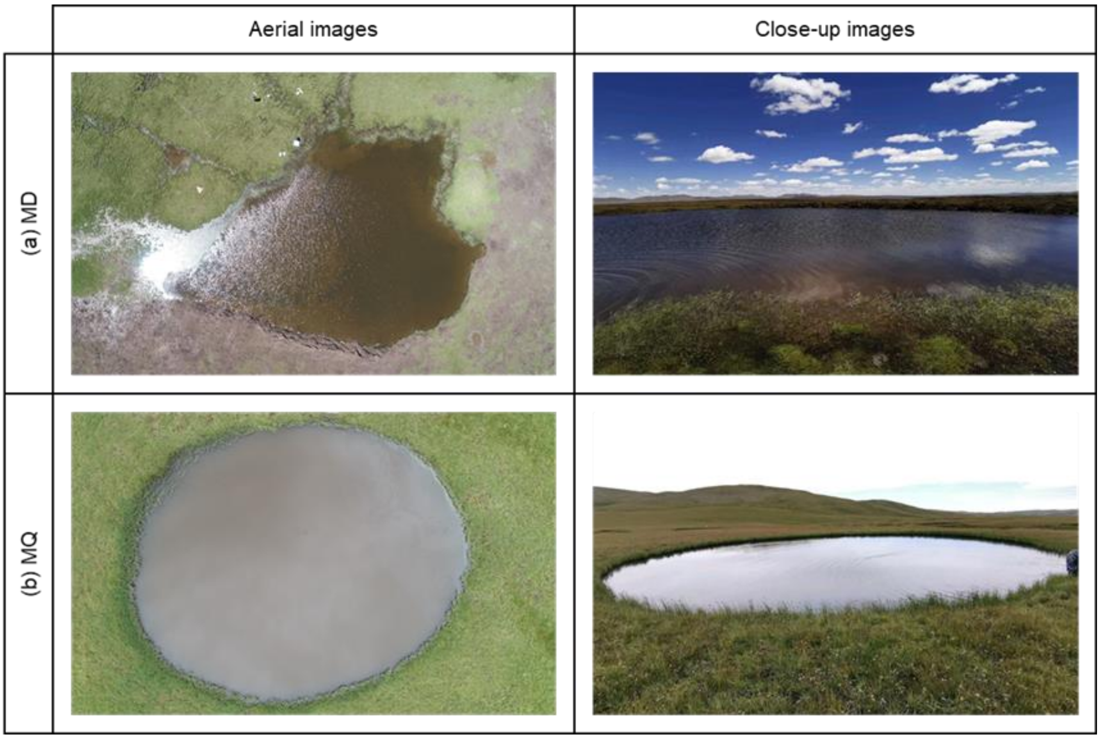

Supplementary Figure 11. Aerial and close-up images of thermokarst lakes on the Tibetan plateau. Aerial images (left) and close-up images (right) of thermokarst lakes in Madoi County (MD, a) and Maqên County (MQ, b) in swamp meadows and alpine meadows. The photographs were captured by Ziliang Li and Luyao Kang in August 2020.

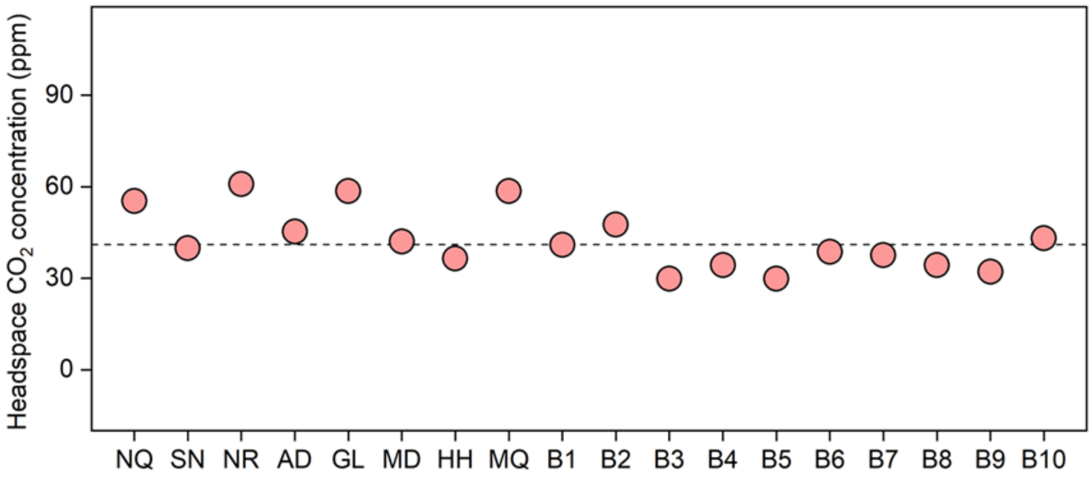

Supplementary Figure 12. The initial headspace carbon dioxide (CO_2) concentrations of the incubated water samples. B1 to B10 represent ten blank samples. The sampling sites are Nagqu (NQ), Seni District (SN), Nyainrong County (NR), Amdo County (AD), Golog Tibetan Autonomous Prefecture (GL), Madoi County (MD), Heihe River (HH) and Maqên County (MQ).

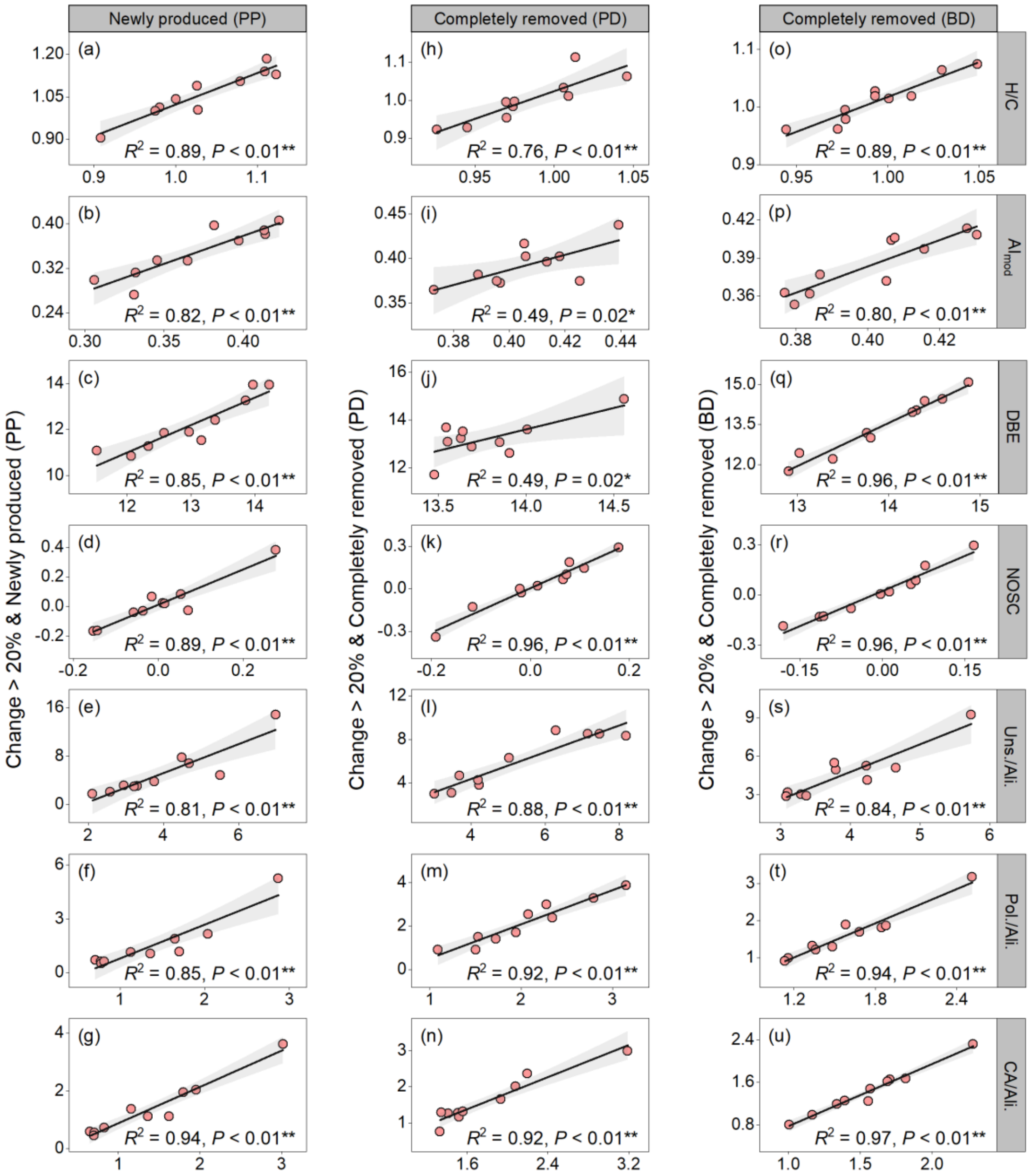

Supplementary Figure 13. Correlation of the properties of photo-produced (PP) (a-g), photo-degraded (PD) (h-n), and bio-degraded (BD) (o-u) compounds between the two calculation methods. The solid line and grey area represent the linear regression line and the 95% confidence interval, respectively. The two-sided statistical tests indicate significant effects by **P* < 0.05; ***P* < 0.01. H/C, the ratio of the number of hydrogen atoms to the number of carbon atoms; AI_{mod}, modified aromaticity index; DBE, the double bond equivalence; NOSC, the nominal oxidation state of carbon;

Uns./Ali., the ratio of highly unsaturated and phenolic compounds to aliphatic compounds; Pol./Ali., the ratio of vascular plant-derived polyphenols to aliphatic compounds; CA/Ali., the ratio of combustion-derived polycyclic aromatics to aliphatic compounds.

Supplementary Table 1. Results of a three-way ANOVA on the effects of light, temperature, and lake location on microbial respiration response to ultraviolet (UV) light exposure (CO_2-RR).

Factor	df	Mean square	*F*-value	*P*-value
Sunlight (S)	1	48.7	3.87	0.05
Temperature (T)	1	33.7	2.68	0.11
Lake location (L)	9	404.1	32.13	<0.001
S×T	1	6.0	0.48	0.49
S×L	9	31.1	2.48	0.02
T×L	9	17.5	1.39	0.21
S×T×L	9	8.1	0.65	0.75

Supplementary Table 2. Microbial cell counts measurements for Milli-Q water, GF/F filtered and 0.22 μm filtered thermokarst lakes water and the latter exposed to simulated sunlight or kept in the dark.

Lake Name	Filter	Experiment Treatment	Microbial cell counts ($\times 10^4$ cells mL^{-1})
NQ	GF/F	/	11
NQ	0.22 μm	/	3
NQ	0.22 μm	Light-exposed 21 hours	3
NQ	0.22 μm	Dark control 21 hours	10
SN	GF/F	/	25
SN	0.22 μm	/	1.7
SN	0.22 μm	Light-exposed 21 hours	2
SN	0.22 μm	Dark control 21 hours	4
GL	GF/F	/	64
GL	0.22 μm	/	1.7
GL	0.22 μm	Light-exposed 21 hours	5
GL	0.22 μm	Dark control 21 hours	21
Milli-Q water	/	/	5.4
Milli-Q water	/	/	4.9
Milli-Q water	/	/	3.7

Notes: microbial cell counts are determined by flow cytometry (Agilent NovoCyte 1040). The sampling sites are Nagqu (NQ), Seni District (SN), and Golog Tibetan Autonomous Prefecture (GL).

Supplementary Table 3. Climate and water properties of 10 thermokarst lakes on the Tibetan alpine permafrost region.

Site	MAP (mm)	MAT (℃)	Salinity (ppt)	Conductivity (mS cm^{-1})	DO (mg L^{-1})	DOC (mg L^{-1})	DIC (mg L^{-1})	DON (mg L^{-1})	pH	a_{300} (m^{-1})	a_{305} (m^{-1})	$SUVA_{254}$ (L mg C^{-1} m^{-1})	S_R	BIX	HIX
YS	306.90	-4.03	0.36	606.67	8.20	6.62	17.64	0.41	8.33	8.44	7.37	1.40	1.29	0.71	0.81
GM	343.60	-2.77	0.25	416.03	4.48	7.37	18.96	0.41	8.34	18.96	17.20	2.35	0.92	0.59	0.88
NQ	444.93	-1.80	0.09	165.13	13.89	8.48	7.53	0.45	8.01	16.89	15.28	1.98	1.19	0.67	0.86
SN	464.90	-0.13	0.25	444.90	6.51	16.29	16.57	0.63	8.22	65.87	60.57	3.45	0.83	0.51	0.94
NR	464.00	-0.83	0.08	140.60	10.15	10.88	7.02	0.61	7.85	32.86	29.86	2.75	0.92	0.61	0.88
AD	456.53	-0.77	0.30	463.70	6.47	11.66	24.04	0.56	8.06	40.65	44.14	3.49	0.95	0.57	0.90
GL	409.03	-1.03	0.21	314.93	4.29	12.16	14.43	1.13	8.06	34.70	31.24	2.54	0.89	0.60	0.92
MD	436.73	-3.17	0.11	169.33	8.10	13.44	9.13	0.81	7.94	39.00	35.39	2.70	0.93	0.55	0.91
HH	373.97	-2.97	0.12	206.77	8.30	5.69	17.48	0.33	8.14	13.74	12.28	2.27	1.03	0.64	0.88
MQ	496.63	-0.87	0.10	147.63	6.56	16.76	7.59	1.35	7.83	54.89	49.90	3.08	0.89	0.56	0.92

Notes: MAP, mean annual precipitation; MAT, mean annual temperature; DO, dissolved oxygen; DOC, dissolved organic carbon; DIC, dissolved inorganic carbon; DON, dissolved organic nitrogen; a_{300} and a_{305}, the Naperian absorption coefficient indicating chromophoric DOC content at 300 nm and 305nm, respectively; $SUVA_{254}$, the absorbance at 254 nm divided by DOC concentration; S_R, slope coefficient ratio correlated to DOC

molecular weight; BIX, biological index; HIX, humification index. The ten sampling sites are Yushu (YS), Golmud (GM), Nagqu (NQ), Seni District (SN), Nyainrong County (NR), Amdo County (AD), Golog Tibetan Autonomous Prefecture (GL), Madoi County (MD), Heihe River (HH), and Maqên County (MQ).

Supplementary Table 4. Extraction efficiency (%) and reproducibility (%) of the styrene divinyl benzene polymer (PPL) column in thermokarst lakes.

Lakes	Treatment	PPL Extraction Efficiency (%)	Number of Common Peaks	Reproducibility (%)
NQ	Dark	61.23	5138	71.51
NQ	Light	86.00	6710	69.17
SN	Dark	56.28	6300	71.49
SN	Light	59.11	6829	73.11
GL	Dark	52.49	8694	75.72
GL	Light	58.67	9538	74.82
Mean (SD)		62.29 (11.99)	/	72.64 (2.41)

Notes: SD, standard deviation. The sampling sites are Nagqu (NQ), Seni District (SN), and Golog Tibetan Autonomous Prefecture (GL).

Supplementary Table 5. List of reanalysis data from satellites as SMARTS model input data and their sources.

Variable	Publication	Data sources
SPR (mb)	Yang et al. (2019)[6]	https://data.tpdc.ac.cn/zh-hans/data/8028b944-daaa-4511-8769-965612652c49/
RH (%)	Wang (2022)[7]	https://www.tpdc.ac.cn/zh-hans/data/99dd84e2-288d-4db8-b098-8118b3b0c17a
TDAY (°C)	National Earth System Science Data Center, National Science & Technology Infrastructure of China	http://www.geodata.cn/datapplication/OrderStepList.html?dataguid=250085273409240
IH2O (cm)	Dee et al. (2011)[8]	https://doi.org/10.1002/qj.828
TAU550	National Earth System Science Data Center, National Science & Technology Infrastructure of China	http://www.geodata.cn/data/datadetails.html?dataguid=1776940&docId=3316

Notes: SPR, surface pressure; RH, relative humidity; TDAY, the average daily temperature at the site level; IH2O (cm), precipitable water; TAU550, aerosol optical depth at 550 nm, τ550.

Supplementary Reference

1. Bowen, J. C., Kaplan, L. A., Cory, R. M. Photodegradation disproportionately impacts biodegradation of semi-labile DOM in streams. *Limnol. Oceanogr.* **65**, 13-26 (2020).
2. Cory, R. M., Ward, C. P., Crump, B. C., Kling, G. W. Sunlight controls water column processing of carbon in arctic fresh waters. *Science* **345**, 925-928 (2014).
3. Gueymard, C. A. *Solar Resources Mapping* Ch. 5 (Springer, Cham, Switzerland, 2019).
4. Gueymard, C. A., Habte, A., Sengupta, M. Reducing Uncertainties in Large-Scale Solar Resource Data: The Impact of Aerosols. *IEEE J. Photovolt.* **8**, 1732-1737 (2018).
5. Zou, D. et al. A new map of permafrost distribution on the Tibetan Plateau. *The Cryosphere* **11**, 2527-2542 (2017).
6. Yang, K., He, J., Tang, W., Lu, H., Qin, J., Chen, Y., Li, X. China meteorological forcing dataset (1979-2018). National Tibetan Plateau/Third Pole Environment Data Center https://doi.org/10.11888/AtmosphericPhysics.tpe.249369.file (2019).
7. Wang, K. Homogeneous grid dataset of Chinese land surface observation (surface solar radiation, surface wind speed, relative humidity and land surface evapotranspiration). National Tibetan Plateau/Third Pole Environment Data Center https://doi.org/10.11888/Atmos.tpdc.272817 (2022).
8. Dee, D. P. et al. The ERA-Interim reanalysis: configuration and performance of the data assimilation system. *Q. J. R. Meteorol. Soc.* **137**, 553-597 (2011).
