# Supplementary material for: Photo-produced aromatic compounds stimulate microbial degradation of dissolved organic carbon in thermokarst lakes
Source: Nat Commun. 2023 Jun 21;14:3681. doi: 10.1038/s41467-023-39432-2 (PMC10284890; doi:10.1038/s41467-023-39432-2)
Supplement: Supplementary file 3 — Reporting Summary [file 41467_2023_39432_MOESM3_ESM.pdf]

nature portfolio

Corresponding author(s): Leiyi Chen

Last updated by author(s): May 22, 2023

Reporting Summary

Nature Portfolio wishes to improve the reproducibility of the work that we publish. This form provides structure for consistency and transparency in reporting. For further information on Nature Portfolio policies, see our Editorial Policies and the Editorial Policy Checklist.

Statistics

For all statistical analyses, confirm that the following items are present in the figure legend, table legend, main text, or Methods section.

n/a	Confirmed	
☐	☒	The exact sample size (*n*) for each experimental group/condition, given as a discrete number and unit of measurement
☐	☒	A statement on whether measurements were taken from distinct samples or whether the same sample was measured repeatedly
☐	☒	The statistical test(s) used AND whether they are one- or two-sided<br>*Only common tests should be described solely by name; describe more complex techniques in the Methods section.*
☒	☐	A description of all covariates tested
☐	☒	A description of any assumptions or corrections, such as tests of normality and adjustment for multiple comparisons
☐	☒	A full description of the statistical parameters including central tendency (e.g. means) or other basic estimates (e.g. regression coefficient) AND variation (e.g. standard deviation) or associated estimates of uncertainty (e.g. confidence intervals)
☐	☒	For null hypothesis testing, the test statistic (e.g. *F*, *t*, *r*) with confidence intervals, effect sizes, degrees of freedom and *P* value noted<br>*Give P values as exact values whenever suitable.*
☒	☐	For Bayesian analysis, information on the choice of priors and Markov chain Monte Carlo settings
☒	☐	For hierarchical and complex designs, identification of the appropriate level for tests and full reporting of outcomes
☐	☒	Estimates of effect sizes (e.g. Cohen's *d*, Pearson's *r*), indicating how they were calculated

Our web collection on statistics for biologists contains articles on many of the points above.

Software and code

Policy information about availability of computer code

Data collection	No software was used for data collection.
Data analysis	All the statistical analyses were performed in R (version 4.0.5). The data from FT-ICR MS was analyzed using Bruker Compass DataAnalysis (version 4.2). The microbial data were analyzed on the Magigene Cloud Platform (http://cloud.magigene.com) using the following software: fastp software (version 0.14.1), cutadapt software (V1.14), usearch (V10), UPARSE (version 7.1), SILVA (v132), and Unite (v8.0). The photon flux was simulated using the NREL SMARTS model (V.2.9.5). Details of these softwares are available in the Methods and supplementary Methods.

For manuscripts utilizing custom algorithms or software that are central to the research but not yet described in published literature, software must be made available to editors and reviewers. We strongly encourage code deposition in a community repository (e.g. GitHub). See the Nature Portfolio guidelines for submitting code & software for further information.

Data

Policy information about availability of data

All manuscripts must include a data availability statement. This statement should provide the following information, where applicable:

- Accession codes, unique identifiers, or web links for publicly available datasets
- A description of any restrictions on data availability
- For clinical datasets or third party data, please ensure that the statement adheres to our policy

Data availability

All prokaryotic and fungal sequences have been deposited in NCBI's SRA database under project accession number PRJNA948140 and PRJNA948167. All data required to reproduce the results are available in the supplementary Table and from the figshare database (https://doi.org/10.6084/m9.figshare.22331278).

Human research participants

Policy information about studies involving human research participants and Sex and Gender in Research.

Reporting on sex and gender	The study did not involve human research participants.
Population characteristics	The study did not involve human research participants.
Recruitment	The study did not involve human research participants.
Ethics oversight	The study did not involve human research participants.

Note that full information on the approval of the study protocol must also be provided in the manuscript.

Field-specific reporting

Please select the one below that is the best fit for your research. If you are not sure, read the appropriate sections before making your selection.

☐ Life sciences ☐ Behavioural & social sciences ☒ Ecological, evolutionary & environmental sciences

For a reference copy of the document with all sections, see nature.com/documents/nr-reporting-summary-flat.pdf

Ecological, evolutionary & environmental sciences study design

All studies must disclose on these points even when the disclosure is negative.

Study description	We conducted a UV light and biodegradation experiment in the lab to explore the light effect on DOC biodegradation across the thermokarst lakes. First, we set up 60 Whirl-Pak bags (10 lakes × 2 light treatments × 3 replicates) in the photo-degradation experiment. After exposure to UV light, subsamples for each light-exposed or dark-control replicate was transferred into a 50 mL amber jar to examine the subsequent biodegradation. After pre-incubation, all the water samples were flushed with CO2-free air for 10 min to homogenize the initial CO2 concentration in the jar and then incubated in dark at 10°C and 20°C for 28 days. In total, 384 microcosms (60Whirl-Pak bags × 2 temperatures × 3 time periods + 24 blanks) were set up to measure microbial CO2 production.
Research sample	The samples in this study represent water collected from 196 thermokarst lakes at 48 sites along an approximately 1100-km permafrost transect across the Tibetan Plateau. To address the cost constraints, a subset of 10 representative thermokarst lakes from the original 48 sites was selected for conducting the sunlight and microbial degradation experiment. Within each selected lake, three replicates were established to perform subsequent UV light and biodegradation experiments. These samples and replicates were chosen to capture the variability and key characteristics of the overall population.
Sampling strategy	The sample size for this study was determined based on several considerations. Firstly, the surface water samples were collected from different positions within each thermokarst lake, including the center, edge, and middle positions from the center to the shore, to capture spatial variability. Additionally, 10 representative thermokarst lakes were selected for the sunlight and microbial degradation experiment. These lakes were chosen to cover a wide range of precipitation gradients, with mean annual precipitation ranging from 307 to 497 mm, and to encompass variations in DOC quantities and composition across the Tibetan Plateau region. Detailed information on DOC, DON, CDOM, and aromatic compounds is provided in Supplementary Dataset 1. Furthermore, to ensure statistical robustness, three replicate samples were run for most variables, such as DOC, nutrient content, CDOM, fluorescent dissolved organic matter, and microbial CO2 respiration, in the UV and biodegradation experiment. This allows for basic statistical analyses and increases the reliability of the results. For the measurement of microbial CO2 respiration, CO2 was measured three times during the incubation period, as described in the manuscript. This approach provides sufficient data points to accurately assess microbial activity. Therefore, the sample sizes were determined based on the need to capture spatial variability, encompass a wide range of environmental conditions, and ensure statistical reliability for the variables under investigation.
Data collection	Field data, including latitude, longitude, elevation, and lake depth, was collected for the study. Additionally, data on sunlight and microbial degradation experiment, such as DOC content and composition, microbial respiration, and community structure, were obtained. The data collection process involved the use of commercial software provided with the respective instruments.
Timing and spatial scale	From July to August 2020, we collected water samples from thermokarst lakes along an about 1100-km permafrost transect across the Tibetan Plateau. We measured initial indicators of the lakes and conducted pre-experiments in September. In October, we first conducted a 21 hours light experiment. Then, the DOC, nutrient content, CDOM, fluorescent dissolved organic matter (FDOM),were measured after UV light experiment. Afterwards, subsampleswas transferred into a 50 mL amber jar and incubated in dark for 28 days at 10°C and 20°C. During this biodegration experiment, microbial CO2 productions were measured on days 0, 7 and 28.
Data exclusions	No data were excluded from the analysis.
Reproducibility	We applied three replicates for each treatment in each lake. All replicates were successful. Given the high cost of FT-ICR MS, DOM molecular composition was only assessed by using the composite sample of the three replicates for each thermokarst lake.

Randomization	The allocation of samples into experimental groups was conducted by randomly selecting UV-transparent Whirl-Pak bags and water bottles for use in the experiment.
Blinding	Blinding was not relevant to this study because it involved objective measurements and experimental procedures that did not rely on subjective judgments or biases. The data collection and analysis were conducted using standardized protocols and automated methods, ensuring objective and unbiased results. Therefore, blinding was not necessary as the study design inherently minimized the potential for observer bias or influence on the outcomes.

Did the study involve field work? ☒ Yes ☐ No

Field work, collection and transport

Field conditions	We collected water samples from thermokarst lakes along an 800-km permafrost transect across the Tibetan Plateau. These thermokarst lakes encompass a wide climatic gradient (Supplementary Dataset 1, mean annual precipitation: 307-497 mm; mean annual temperature: -4.03 to -0.13°C).
Location	We selected 10 representative thermokarst lakes with a longitude range of 91.81-99.33E and a latitude range of 31.30-34.83N (Supplementary Fig. 1). These lakes are distinct in altitude (spanning of 4185m to 5080m) and depth (ranging from 24m to 68 m).
Access & import/export	Access were determined by the three-river-source national park (July 10th to August 25th, 2020) and Qinghai Hoh Xil National Natural Reserve (July 10th to August 25th, 2020).
Disturbance	Samples near the lakes were taken by simple devices, and samples in the center of lakes were taken by rowing. These efforts were done to minimize the disturbulence on the samples and the lake ecosystem.

Reporting for specific materials, systems and methods

We require information from authors about some types of materials, experimental systems and methods used in many studies. Here, indicate whether each material, system or method listed is relevant to your study. If you are not sure if a list item applies to your research, read the appropriate section before selecting a response.

Materials & experimental systems

n/a	Involved in the study
☒	☐ Antibodies
☒	☐ Eukaryotic cell lines
☒	☐ Palaeontology and archaeology
☒	☐ Animals and other organisms
☒	☐ Clinical data
☒	☐ Dual use research of concern

Methods

n/a	Involved in the study
☒	☐ ChIP-seq
☒	☐ Flow cytometry
☒	☐ MRI-based neuroimaging
